# Evaluation of cut-off values in acute paracetamol overdose following the United Kingdom guidelines

**DOI:** 10.1186/s40360-021-00547-1

**Published:** 2022-01-05

**Authors:** Hyun Ho Jeong, Kyungman Cha, Kyoung Ho Choi, Byung Hak So

**Affiliations:** 1 Department of Emergency Medicine, Uijeongbu St. Mary’s Hospital, College of Medicine, The Catholic University of Korea, Seoul, Republic of Korea; 2 Department of Emergency Medicine, Suwon St. Vincent Hospital, College of Medicine, The Catholic University of Korea, Seoul, 93 Jungbu Blvd., Paldal, Suwon, Gyeonggi Republic of Korea 16247

**Keywords:** Paracetamol, Overdose, Acetylcysteine, Antidote

## Abstract

**Background:**

The United Kingdom guideline for acute paracetamol overdose has recommended the use of ‘100-treatment line’. Emergency medical centers in some developing countries lack the resources for timely reporting of paracetamol concentrations, hence treatment depends on reported dose. This study aimed to examine whether using an reported dose is safe to predict concentration above the 100-line.

**Methods:**

Data were retrieved from two emergency medical centers retrospectively, between 2010 and 2017. The inclusion criteria were single acute paracetamol overdose, presentation within 15 h, and age ≥ 14 years. Multiple linear regression was performed to determine the effect of ingested dose on paracetamol concentration. Subgroups were created based on ingested dose, rate of concentration above 100-line were investigated.

**Results:**

One hundred and seventy-two patients were enrolled in the primary analysis; median dose was 133.3 mg/kg and 46 (37.8%) had concentration above 100-line in the first test. Only dose per weight was moderately correlated with the first concentration (R^2^ = 0.410, *p* < 0.001). In the ≤200 mg/kg ingestion group, 18 patients showed concentration above 100-line and 8 showed acute liver injury. The cut-off value of 150 mg/kg showed 82.6% sensitivity and 73.8% specificity to predict concentration above 100-line.

**Conclusion:**

Where paracetamol concentration is not available and activated charcoal is readily used, following United Kingdom guideline, it is safe to use an ingested dose of > 150 mg/kg as the cut-off value for N-acetylcysteine treatment with risk stratification for hepatotoxicity if the patient is ≥14 years and visit the ED within 15 h after an acute paracetamol overdose.

**Supplementary Information:**

The online version contains supplementary material available at 10.1186/s40360-021-00547-1.

## Background

The risk prediction of hepatotoxicity in acute paracetamol (APAP) overdose has relied on the Prescott nomogram since the 1970s. The nomogram utilizes time of ingestion and serum APAP concentration to guide the need for N-acetylcysteine (NAC) treatment [1]. However, the nomogram treatment line indicating NAC therapy differs from country to country [2, 3].

The serum APAP level that can be reported while treating a patient is key to successfully managing acute APAP overdose. However, poison centers and emergency medical centers in some developing countries lack the laboratory resources required for timely reporting of drug concentrations. Hence, whether NAC is administered in cases of acute APAP overdose depends almost entirely on routine laboratory test results and the dose of APAP reported by the patient [4]. Although patient-reported dose is a strong predictor of hepatotoxicity and the need for NAC treatment [5–7], careful and detailed collection of medical history and information from patients and guardians is necessary to determine the actual ingested dose. Determining NAC treatment based on the reported dose is inevitably limited when NAC is administered before concentration measurement for patients who have ingested very high doses; it is also controversial when the ingested dose is thought to be lower than 200 mg/kg in an environment where APAP concentration is not available [6, 7].

Most countries, including the United States, Canada, Australia, Singapore, and South Korea, have used the 150-treatment line (150 μg/mL at 4 h and 37.5 μg/mL at 12 h post-ingestion) to treat patients with single acute APAP poisoning for over 30 years; however, the UK and a few countries have used the 100-treatment line (100 μg/mL at 4 h and 25 μg/mL at 12 h) for patients with hepatotoxicity-related risk factors [8, 9]. In 2012, the Medicines and Healthcare products Regulatory Agency and the Commission on Human Medicines in the UK expanded this guideline to include patients who ingest more than the maximum therapeutic dose of APAP (75 mg/kg body weight in 24 h) [10–12]. However, because the institutions that participated in the study could not get APAP concentration during treatment, NAC antidote therapy has been commenced for all patients presumed to have ingested more than 200 mg/kg or 10 g in total.

The present study aimed to examine whether using the ingested dose of APAP is safe to predict APAP concentration above the 100-treatment line following the UK guideline in settings where the serum APAP concentration cannot be reported expeditiously.

## Methods

### Study setting and design

The data of patients who presented to two emergency medical centers in Seoul and Suwon, the Republic of Korea, between January 1, 2010, and December 31, 2017, were retrieved from the toxicology registers of the centers. The emergency centers were in urban academic hospitals that managed > 60,000 patients annually. The data were recorded by the chief emergency physician on duty using a digitally standardized form on patient presentation.

### Study population

The inclusion criteria were single acute APAP overdose, emergency department (ED) visitation within 15 h after the overdose, and age ≥ 14 years. The exclusion criteria were staggered ingestion over 1 h, ingestion of extended-release tablets, and unavailability of data on recorded body weight, the ingested dose, or APAP concentration.

### Data collection

The data collected included patient demographics (e.g., age, sex, and weight), the reported ingested dose of APAP and time of ingestion, time of presentation to the ED, intentionality, composition of APAP, co-ingested substances, underlying hepatic disease, alcohol consumption, drug history, treatment methods (e.g., gastric lavage, activated charcoal (AC), and N-acetylcysteine (NAC)), laboratory test results, serum APAP concentration at time, and clinical outcome.

The highest dose of ingested APAP, as estimated from information provided by the patient, his/her guardians, and the emergency services, was used as the ingested dose, and the longest time from ingestion to presentation was also judged as the elapsed time. Staggered ingestion was defined as multiple APAP doses (including supratherapeutic doses) over a > 1 h period, and acute starvation was defined as a state of having suffered a debilitating problem such as receiving treatment for an eating disorder. Co-ingested substances were recorded if they were noted by the patient, identified through the remaining medicines, hospital prescriptions, or by contacting other hospitals. Chronic alcohol consumption was defined as the ingestion of > 14 standard alcohol doses per week, and acute liver injury was defined as alanine aminotransferase (ALT) elevation ≥50% during treatment; hepatotoxicity was defined as ALT elevation > 1000 IU/L. The time for APAP concentration was recorded in minutes from the sampling time in the test result report. If a test was performed before 240 min (4 h) from the overdose onset, assuming the margin of error to be 5%, tests within 12 min were regarded as being performed at 240 min, and tests outside this range were not accepted, and in this case, the next test was regarded as the first test.

Two investigators separately reviewed the registry, and a third investigator independently checked the data and corrected mismatched variables. All three investigators were medical personnel in the ED.

Antidote therapy consisting of intravenous (IV) NAC infusion for 21 h was initiated when the estimated dose of APAP exceeded 200 mg/kg/24 h or ≥ 10 g in total, the ingestion was staggered, or the ingestion time was uncertain. The 21-h IV NAC protocol required IV loading of 150 mg/kg for 15 min, 45 min later, IV infusion of 50 mg/kg for 4 h and 100 mg/kg for 16 h. Blood samples for the first serum APAP concentration were obtained at least 4 h after ingestion, and subsequent tests were performed every 4 h. The participating emergency medical centers lacked laboratory facilities for timely reporting of serum APAP concentrations. Therefore, sample analysis was outsourced to professional clinical laboratory agencies (Seoul Clinical Laboratories, Yongin, Republic of Korea and Samkwang Medical Laboratories, Seoul, Republic of Korea), with the test results confirmed later. The diagnostic systems used by these agencies were the Cobas® 8000 and Cobas® Integra 400 plus (Roche Diagnostics, Mannheim, Germany), respectively.

### Statistical analysis

Continuous variables were reported as means if they followed a normal distribution or medians if they did not. Categorical variables were reported as proportions. The concentration ratio (the first concentration divided by the nomogram concentration at the same time in minutes) was calculated to determine concentrations above the 100-treatment line. The χ^2^ and Mann–Whitney U tests were used to compare the proportion and distribution of variables between the APAP concentration above-line and under-line groups.

To determine the effect of the ingested dose per weight on APAP serum concentration, a simple linear regression was performed. Using multiple linear regression, variables with variance inflation factor (VIF) greater than 4.0 were excluded from subsequent regression. The correlation between the risk factors used to indicate the need for NAC treatment before the guideline was revised and concentrations above the 100-treatment line were evaluated using univariate and multivariate logistic regression. The odds ratios (OR) and 95% confidence interval (CI) were estimated.

Three subgroups were created based on the reported dose per body weight: the ≤75 mg/kg, 75–200 mg/kg, and > 200 mg/kg ingestion groups, and the occurrence rate of concentration above 100-line and laboratory abnormality of the subgroups were investigated. The area under the receiver operating characteristic curve (AUC) of poisoning dose per body weight for predicting the concentration above the 100-treatment line was calculated. (SPSS version 22.0 software, IBM Corp., Armonk, NY, USA).

## Results

### Study population

During the study period, 373 patients visited the ED owing to acute APAP overdose. Patients were excluded if they were < 14 years of age (*n* = 16), presented to the ED > 15 h after overdose (*n* = 32), had staggered ingestion (*n* = 9), ingested extended-release tablets (*n* = 74), and had unrecorded ingested doses (*n* = 18), weight information (*n* = 59) or APAP concentration (*n* = 30). Thus, 172 patients were enrolled in the primary analysis.

Twenty-nine patients (16.9%) were men, and 143 (83.1%) were women. The median age was 23 years (interquartile range [IQR], 17–38), and the median weight was 57.0 kg (IQR, 50.0–63.0). One hundred sixty cases (93.0%) involved intentional self-harm attempts. One hundred forty-one cases (82.0%) ingested APAP with other classes of medications, and 33 of them overdosed with substances that delayed gastric emptying or activated hepatic enzymes: this included scopolamine, pheniramine, chlorpheniramine, diphenhydramine, dimenhydrinate, methylphenidate, cetirizine, levocetirizine, codeine, dihydrocodeine, dextromethorphan, chlorzoxazone, levodopa, and carbidopa (Table 1).

**Table 1:** Demographic and clinical characteristics of the patients with serum APAP concentration above or under 100-treatment line

		Under 100-line (*n* = 126)	Above 100-line (*n* = 46)	*p*-value
Gender, Male	29 (16.9)	22 (17.5)	7 (15.2)	0.728
Age (year)	23 (17–38)	24 (17–37)	22 (18–38)	0.683
Intentionality	160 (93.0)	116 (92.1)	44 (95.7)	0.414
Weight (kg)	57.0 (50.0–63.0)	57.0 (50.0–63.0)	56.5 (46.0–60.0)	0.220
Total ingested dose (g)	7.7 (5.0–12.0)	6.0 (4.9–10.0)	10.0 (6.0–16.5)	< 0.001
Ingested dose per kilogram of weight (mg/kg)	133.3 (88.5–199.3)	106.5 (80.0–153.1)	248.1 (158.3–391.3)	< 0.001
Time from ingestion to presentation (minute)	172 (77–332)	139 (67–309)	241 (145–468)	0.002
Time from ingestion to administration of activated charcoal (minute)^a^	152 (85–271)	117 (76–221)	207 (123–364)	0.003
Acute starvation	9 (5.2)	6 (4.8)	3 (6.5)	0.646
Chronic liver disease	1 (0.6)	0 (0.0)	1 (2.2)	0.097
Chronic alcohol consumption	14 (8.1)	10 (7.9)	4 (8.7)	0.872
Co-ingestion^b^	33 (19.2)	26 (20.6)	7 (15.2)	0.424
Activated charcoal^a^	120 (69.8)	90 (71.4)	30 (65.2)	0.432
N-acetylcysteine treatment	131 (76.2)	86 (68.3)	45 (97.8)	< 0.001
Albumin (g/dL)	4.6 (4.4–4.8)	4.6 (4.4–4.8)	4.5 (4.3–4.8)	0.464
Acute liver injury	9 (5.2)	7 (5.6)	2 (4.3)	0.753

Table 1. Variables are expressed as n (%) or median (interquartile range).

^a^ *n* = 120

^b^ Co-ingestion: overdose with substances that delayed gastric emptying or induced hepatic enzymes

**Fig. 1:**
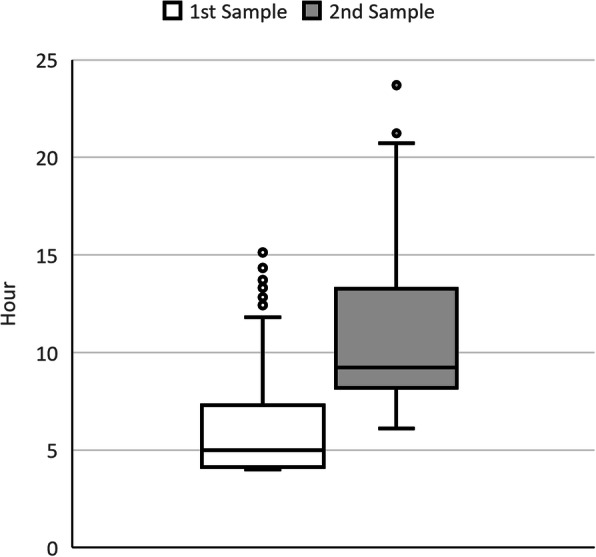
Time from overdose to paracetamol concentration test

Forty-six patients (37.8%) had serum APAP concentrations above the 100-treatment line in the first test. The median time from ingestion to the first test for APAP serum concentration was 300 min (IQR, 247–437), and the time to the second test was 553 min (IQR, 491–796; *n* = 129) (Fig. 1). One hundred and twenty patients (69.8%) were treated with AC. Among the 62 patients (36.0%) who had ingested > 10 g or > 200 mg/kg of APAP, all but one (who refused treatment) received NAC therapy.

### The correlation of ingested dose per weight and risk factors for hepatotoxicity with concentrations above the 100-treatment line

**Fig. 2:**
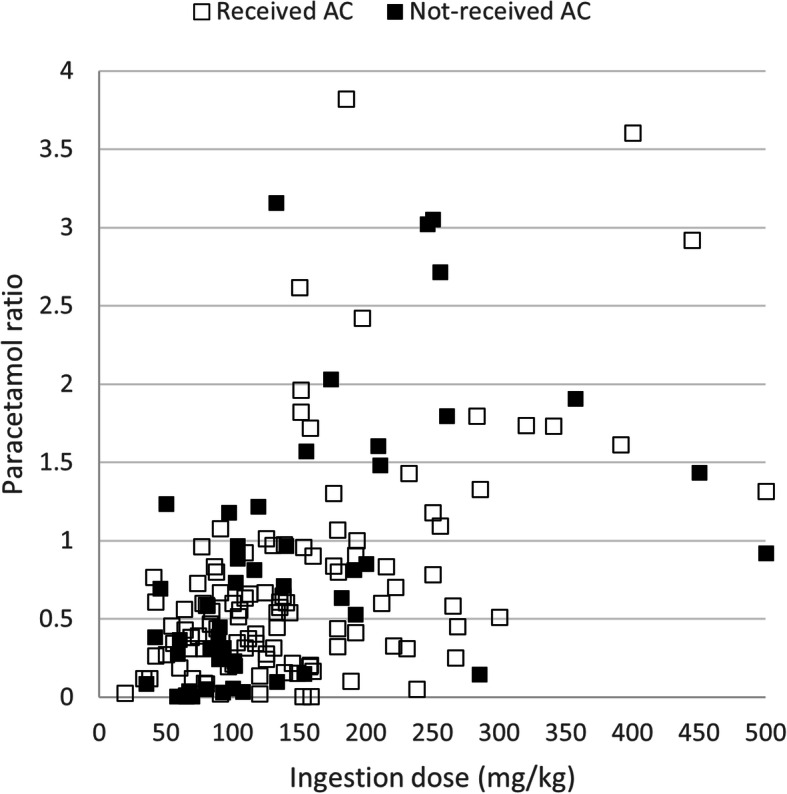
The relationship between paracetamol ratio and ingestion dose per weight

The ingested dose per weight was moderately correlated with the first APAP serum concentration and the ratio of concentration in the simple linear regression analysis (r = 0.603, *p* < 0.001; r = 0.513, *p* < 0.001) (Fig. 2).

In the multiple linear regression analysis, total ingested dose, and elapsed time from overdose to the administration of AC showed collinearity for the first APAP concentration; thus, they were excluded from subsequent regression (VIF = 63.740 and 78.056) (Supplement 1). Finally, only ingested dose per weight was moderately correlated with the first concentration (R^2^ = 0.410, *p* < 0.001).

Of the risk factors, ingested dose per weight and elapsed time from poisoning to ED presentation hardly showed a statistically significant difference in terms of concentration above the 100-line in the logistic regression analysis (OR = 1.008, 95% CI 1.005–1.012; *p* < 0.001, OR = 1.002, 95% CI 1.000–1.004; *p* = 0.013) (Table 2).

**Table 2:** Logistic regression analysis for APAP concentration above 100-line

	Univariate		Multivariate	
	OR (95% CI)	*p*-value	OR (95% CI)	*p*-value
Ingested dose per kilogram of weight (mg/kg)	1.008 (1.005–1.012)	< 0.001	1.008 (1.005–1.012)	< 0.001
Time from ingestion to presentation (minute)	1.002 (1.000–1.004)	0.015	1.002 (1.000–1.004)	0.013

*OR* Odds ratio, *CI* Confidence interval

### Subgroup analysis

**Fig. 3:**
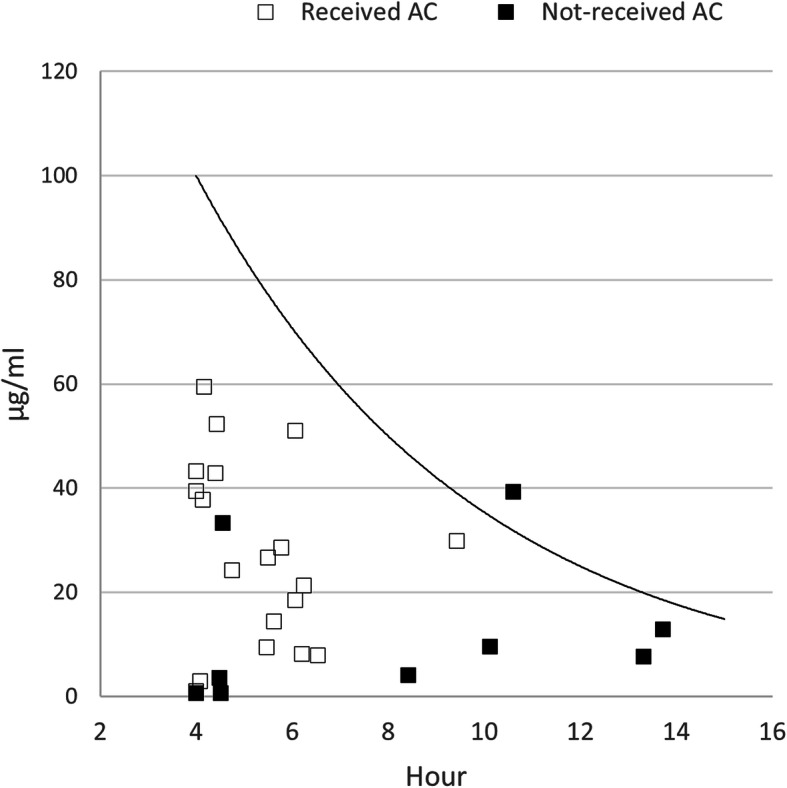
The first paracetamol concentration in the group less than 75 mg per kg

**Fig. 4:**
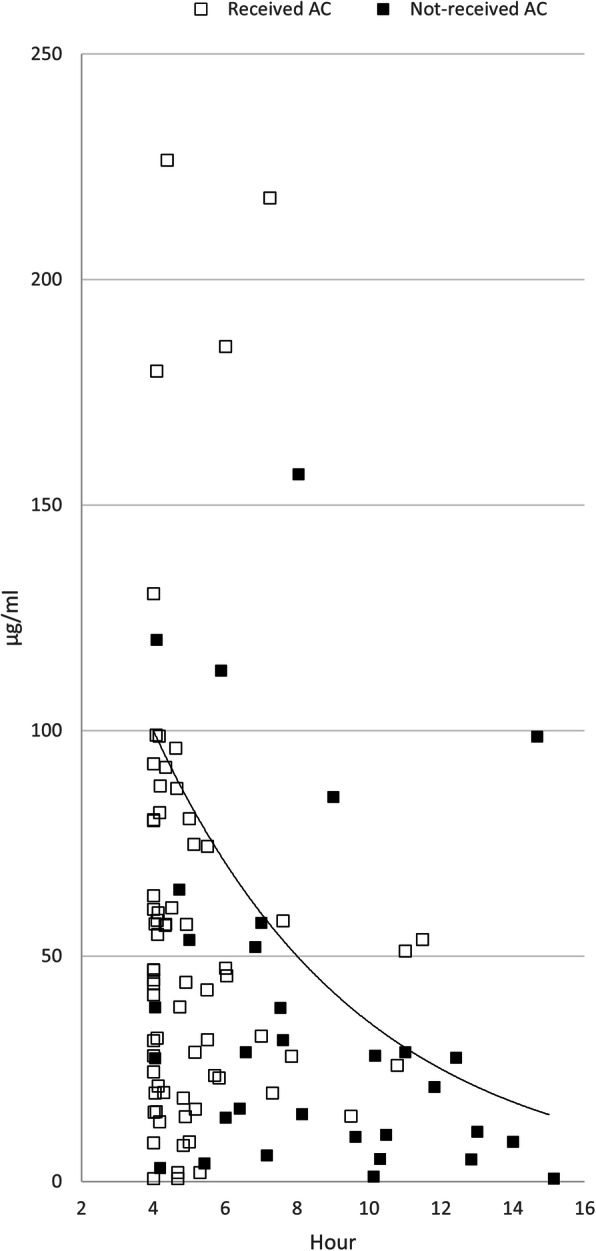
The first paracetamol concentration in the group between 75 and 200 mg per kg

**Fig. 5:**
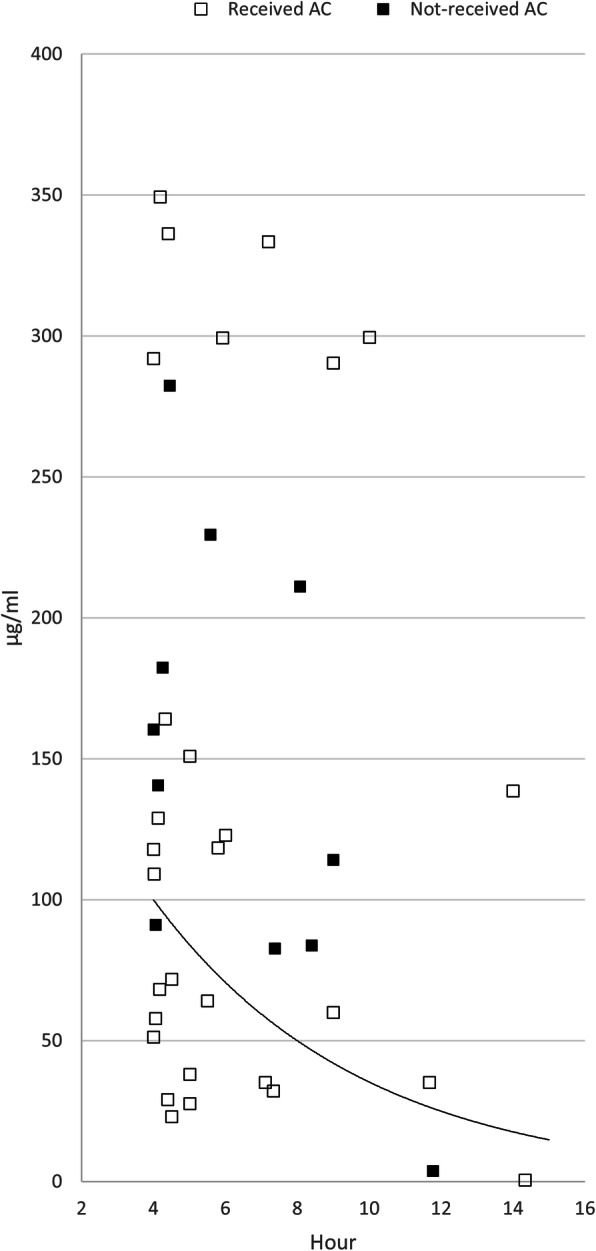
The first paracetamol concentration in the group more than 200 mg per kg

Three subgroups were created based on the dose of APAP ingested per body weight. The proportions of patients with serum APAP concentration above the 100-line were 3.6% (one of 28), 16.7% (17 of 102), and 66.7% (28 of 42) in the ≤75, 75–200, and > 200 mg/kg ingestion groups (Table 3) (Figs. 3, 4, and 5).

**Table 3:** APAP concentration above treatment line and laboratory abnormality of the subgroups based on the ingested dose per body weight

	≤75 mg/kg (*n* = 28)	75–200 mg/kg (*n* = 102)	> 200 mg/kg (*n* = 42)
Above 100-line	1 (3.6)	17 (16.7)	28 (66.7)
Above 150-line	0 (0.0)	10 (9.8)	24 (54.1)
Acute liver injury^a^	2 (7.1)	6 (5.9)	1 (2.4)
Increased INR^b^	0 (0.0)	1 (0.98)	0 (0.0)
Increased creatinine^c^	0 (0.0)	1 (0.98)	1 (2.4)

Table 3. Variables are expressed as n (%) or median (interquartile range). *INR* International normalized ratio.

^a^ Acute liver injury: Alanine aminotransferase elevation ≥50% during treatment

^b^ Increased INR: INR elevation ≥50% during treatment

^c^ Increased creatinine: creatinine elevation ≥50% during treatment

In the 75–200 mg/kg ingestion group, the second APAP concentration was above the 100-treatment line in 3 patients (line crossers), and their respective ingested doses were 138.5 mg/kg, 191.2 mg/kg, and 137.5 mg/kg. The AUC of the ingested dose per weight for the 100-treatment line was 0.851 (95% CI 0.789–0.901, *p* < 0.001), and sensitivity and specificity of the 148.51 mg/kg cut-off value were 84.78% (71.1–93.7) and 73.81% (65.2–81.2).

## Discussion

Previous studies have shown that the dose of APAP reported by the patient can predict hepatotoxicity and the need for NAC antidote therapy, but there has been controversy on the use of the ingested dose when the APAP concentration cannot be determined in time [5–7, 13]. In the study population, the ingested dose per weight was just moderately related with the first APAP concentration (r = 0.603, *p* < 0.001; R^2^ = 0.410, *p* < 0.001), but hardly predictive of concentrations above the 100-line (OR = 1.008, 95% CI 1.005–1.012, *p* < 0.001).

Most of the patients in our study (*n* = 120, 69.8%) were treated with AC. In the group that did not receive AC treatment (*n* = 52), the ingested dose per weight could not accurately predict the first APAP concentration above the 100-line (OR = 1.011, 95% CI 1.003–1.019; *p* = 0.006), with only a moderate correlation (R^2^ = 0.490, *p* < 0.001) (Supplement 2). These statistical results suggest that AC administration did not seem to alter the first APAP concentration; however, it is likely that AC was presumably used in the above group. If so, this underscores the importance of using AC in an environment where APAP concentration is not readily available.

It has been claimed that selecting patients for antidote therapy should be according to health care cost-effectiveness [14, 15]. It is also true that the revised UK guideline is used only in some European countries [14, 16]. In EDs that participated in this study, the cost of a two-day hospital stay was approximately £150–300. If 148.51 mg/kg (cut-off value in the receiver operating characteristic curve) is taken as the cut-off ingested dose, only 30 (17.4%) more patients would be included from the study population, but if 75 mg/kg is taken, the number of additional patients increases to 101 (58.7%), and this huge cost could have a significant impact on the health care budget.

In the ≤200 mg/kg ingestion group (*n* = 130, 75.6%), 18 patients showed first APAP concentrations above the 100-treatment line. One patient of them ingested 50 mg/kg in an acute starvation status, 6 visited ED 8 h after the overdose, 1 had chronic liver disease, 2 were chronic alcoholics, and 3 ingested APAP with substances that delayed gastric emptying or induced hepatic enzymes. Eight patients of these patients showed acute liver injury, and the ALT level in the patient with the highest level was 442 IU/L. Three of these patients exhibited the line-crossing phenomenon; one presented to the ED 8 h after the overdose and could not receive NAC treatment, one overdosed with dextromethorphan, which can delay gastric emptying, and one showed acute liver injury (maximal ALT level, 442 IU/L). Therefore, in this study population, following the revised UK guideline, it may be useful, safe, and cost-effective to use a dose of 148.51 mg/kg or 150 mg/kg (82.6% sensitivity, 73.8% specificity) as the cut-off value for NAC treatment when the patient has a risk factor for hepatotoxicity.

The time from poisoning to ED presentation showed OR of 1.002 (1.000–1.004, *p* = 0.013) in logistic regression for the concentration above 100-line; however, was not statistically significant in multiple linear regression (Unstandardized Coefficients = 0.072, 95% CI -0.036–0.181, *p* = 0.189). This statistical result is considered because the frequency of AC administration was lower in patients who arrived late to the ED than in those who arrived early (χ^2^ = 6.262, *p* = 0.012, Spearman coefficient = − 0.416 in a linear-by-linear association analysis) and explained by collinearity between time from overdose to AC administration and ED presentation (152 min (85–271) and 172 min (77–332); VIF = 78.056 and 80.602).

Blood samples obtained from patients were refrigerated at 4 °C in plain tubes immediately after sampling and collected every weekday afternoon by the testing agencies. Hence, the length of the storage period may have differed by as much as 72 h. According to the information provided by the manufacturer of the diagnostic analysis systems, the samples remain stable for up to 7 days at 2–8 °C in plain tubes [17]. Therefore, it is unlikely that differences in the duration of storage significantly affected serum APAP concentrations.

Fifty-nine and 18 patients were not included in our study owing to the absence of data on body weight and ingested dose, respectively. This information apparently was not recorded because the ingested APAP dose was very low; hence, NAC treatment or concentration test was not required. When calculated using the median body weight value (57.0 kg), 22 patients could be in the ≤75 mg/kg ingestion group, and 26 patients could be in the 75–200 mg/kg ingestion group. Thirty patients excluded due to missing concentration records were in the ≤75 mg/kg ingestion group. This suggests that the occurrence rate of concentrations above the 100-line in patients who ingested relatively low amounts of APAP may be exaggerated.

This research was conducted based on data derived from East Asians in a single country. East Asians have been shown to absorb APAP faster and be less susceptible to liver injury than Caucasians. Accordingly, first-test drug concentrations might have been higher, and the incidence of a delayed increase in serum APAP concentrations is assessed lower [18–20].

This study was a retrospective observational study and subject to selection bias and data entry errors when the patient information was missing, or the medical records were incomplete. Our sample size was small (*n* = 172), the ingested dose was relatively low (median 7.7 g), and a high proportion of patients (69.8%) received AC treatment. Therefore, the proportion of APAP concentration above the 100-treatment line might have been low in the patients who ingested less than 200 mg/kg.

## Conclusion

In settings where serum APAP concentrations cannot be measured expeditiously and AC is actively used, following the revised UK guideline, it is safe, and cost-effective to use a dose of > 150 mg/kg as the cut-off value for NAC treatment with risk stratification for hepatotoxicity if the patient is ≥14 years old and had visited the ED within 15 h after an acute APAP overdose.

## Supplementary Information

**Additional file 1.**

**Additional file 2.**

## Data Availability

The datasets used and/or analyzed during the study are available from the corresponding author upon reasonable request.

## Abbreviations

APAP: paracetamol
AUC: Area Under the Receiver Operating Characteristic Curve
CI: Confidence Interval
ED: Emergency Department
IQR: Interquartile Range
IV: intravenous
KGCP: Good Clinical Practice in the Republic of Korea
NAC: N-Acetylcysteine
OR: Odds Ratio
UK: United Kingdoms
US: United States
