# Supplementary material for: Evaluation of cut-off values in acute paracetamol overdose following the United Kingdom guidelines
Source: BMC Pharmacol Toxicol. 2022 Jan 5;23:5. doi: 10.1186/s40360-021-00547-1 (PMC8734297; doi:10.1186/s40360-021-00547-1)
Supplement: Supplementary file 1 — Additional file 1. [file 40360_2021_547_MOESM1_ESM.docx]

Supplement 1-1. Multicollinearity between the variables on the first APAP concentration.

|  | Unstandardized Coefficients (95% CI) | *p*-value | VIF |
| --- | --- | --- | --- |
| Intentionality | 28.502 (-33.564~90.568) | 0.365 | 1.113 |
| Weight (kg) | 0.654 (-1.302~2.611) | 0.509 | 2.691 |
| Total ingested dose (g) | -0.004 (-0.014~0.006) | 0.426 | 63.740 |
| Ingested dose per kilogram of weight (mg/kg) | 0.513 (-0.060~1.086) | 0.079 | 63.521 |
| Time from ingestion to presentation (minute) | 0.122 (-0.726~0.970) | 0.777 | 80.602 |
| Time from ingestion to the first concentration test (minute) | -0.151 (-0.323~0.020) | 0.083 | 2.367 |
| Time from ingestion to administration of activated charcoal (minute)^a^ | -0.004 (-0.828~0.820) | 0.992 | 78.056 |
| Acute starvation | -7.883 (-78.784~62.981) | 0.826 | 1.055 |
| Chronic alcohol consumption | 19.012 (-32.295~70.319) | 0.464 | 1.058 |
| Co-ingestion^b^ | -39.374 (-73.270~-5.478) | 0.023 | 1.054 |
| Activated charcoal^a^ | -117.946 (-447.190~211.298) | 0.479 | 4.712 |
| Albumin (g/dL) | -18.768 (-49.656~12.120) | 0.231 | 1.100 |

Supplement 1-1. Coefficient of determination, R^2^=0.430 (*p*<0.001) (n=120). Chronic liver disease was excluded due to missing value of correlation coefficient. *CI* Confidence interval, *VIF* Variance inflation factor.

^a^ n=120.

^b^ Co-ingestion: overdose with substances that delayed gastric emptying or induced hepatic enzymes.

Supplement 1-2. Multicollinearity between the variables on the first APAP concentration.

|  | Unstandardized Coefficients (95% CI) | *p*-value | VIF |
| --- | --- | --- | --- |
| Intentionality | 16.682 (-26.213~59.577) | 0.444 | 1.077 |
| Weight (kg) | -0.041(-0.992~0.910) | 0.933 | 1.046 |
| Ingested dose per kilogram of weight (mg/kg) | 0.300 (0.236~0.364) | <0.001 | 1.072 |
| Time from ingestion to presentation (minute) | 0.072 (-0.036~0.181) | 0.189 | 4.353 |
| Time from ingestion to the first concentration test (minute) | -0.137 (-0.265~-0.008) | 0.037 | 4.132 |
| Acute starvation | -5.657 (-56.460~45.145) | 0.826 | 1.033 |
| Chronic liver disease | 46.980 (-93.885~187.845) | 0.511 | 1.035 |
| Chronic alcohol consumption | 10.241 (-28.949~49.431) | 0.606 | 1.036 |
| Co-ingestion^a^ | -24.659 (-51.812~2.495) | 0.075 | 1.029 |
| Activated charcoal | 4.764 (-22.244~31.772) | 0.728 | 1.367 |
| Albumin (g/dL) | -14.826 (-38.210~8.557) | 0.212 | 1.038 |

Coefficient of determination, R^2^=0.410 (*p*<0.001) (n=170). *CI* Confidence interval, *VIF* Variance inflation factor.

^a^ Co-ingestion: overdose with substances that delayed gastric emptying or induced hepatic enzymes.
