# Supplementary material for: Evaluation of cut-off values in acute paracetamol overdose following the United Kingdom guidelines
Source: BMC Pharmacol Toxicol. 2022 Jan 5;23:5. doi: 10.1186/s40360-021-00547-1 (PMC8734297; doi:10.1186/s40360-021-00547-1)
Supplement: Supplementary file 2 — Additional file 2. [file 40360_2021_547_MOESM2_ESM.docx]

Supplement 2-1. Demographic and clinical characteristics of the patients who did not receive AC treatment

|  |  | Under 100-line (n=36) | Above 100-line (n=16) | *p*-value |
| --- | --- | --- | --- | --- |
| Gender, Male | 8 (15.4) | 5 (13.9) | 3 (18.8) | 0.654 |
| Age (year) | 25 (19-39) | 24 (19-34) | 26 (19-43) | 0.507 |
| Intentionality | 47 (90.4) | 32 (88.9) | 15 (93.8) | 0.583 |
| Weight (kg) | 57.0 (50.0-60.0) | 56.0 (50.0-60.0) | 58.5 (46.0-62.5) | 0.960 |
| Total ingested dose (g) | 6.0 (4.9-10.0) | 5.3 (4.4-8.0) | 10.3 (7.3-14.9) | <0.001 |
| Ingestion dose per kilogram of weight (mg/kg) | 105.6 (89.2-192.0) | 96.7 (80.6-135.6) | 209.9 (135.1-258.2) | <0.001 |
| Time from ingestion to presentation (minute) | 334 (175-565) | 327 (133-584) | 352 (203-489) | 0.992 |
| Acute starvation | 4 (7.7) | 2 (5.6) | 2 (12.5) | 0.386 |
| Chronic liver disease | 1 (1.9) | 0 (0.0) | 1 (6.3) | 0.130 |
| Chronic alcohol consumption | 4 (7.7) | 3 (8.3) | 1 (6.3) | 0.795 |
| Co-ingestion^a^ | 6 (11.5) | 4 (11.1) | 2 (12.5) | 0.885 |
| N-acetylcysteine treatment | 34 (65.4) | 19 (52.8) | 15 (93.8) | 0.004 |
| Albumin (g/dL) | 4.6 (4.2-4.8) | 4.6 (4.4-4.8) | 4.5 (4.1-4.8) | 0.542 |
| Acute liver injury | 4(7.7) | 2 (5.6) | 2 (12.5) | 0.386 |

Supplement 2-1. Variables are expressed as n (%) or median (interquartile range)

^a^ Co-ingestion: overdose with substances that delayed gastric emptying or induced hepatic enzymes.

Supplement 2-2. Multicollinearity between the variables on the first APAP concentration of the patients who did not receive AC treatment

|  | Unstandardized Coefficients (95% CI) | *p*-value | VIF |
| --- | --- | --- | --- |
| Intentionality | -9.955 (-68.581~48.670) | 0.733 | 1.296 |
| Weight (kg) | -0.328 (-3.096~2.439) | 0.812 | 3.762 |
| Total ingested dose (g) | 0.000 (-0.010~0.009) | 0.933 | 21.581 |
| Ingestion dose per kilogram of weight (mg/kg) | 0.405 (-0.225~1.035) | 0.201 | 17.489 |
| Time from ingestion to presentation (minute) | 0.057 (-0.106~0.219) | 0.485 | 6.660 |
| Time from ingestion to the first concentration test (minute) | -0.124 (-0.319~0.070) | 0.204 | 6.504 |
| Acute starvation | -37.595 (-108.481~33.292) | 0.290 | 1.187 |
| Chronic liver disease | 43.732 (-71.578~159.043) | 0.448 | 1.090 |
| Chronic alcohol consumption | -12.744 (-72.836~47.349) | 0.670 | 1.113 |
| Co-ingestion^a^ | 27.219 (-25.043~79.482) | 0.299 | 1.209 |
| Albumin (g/dL) | -9.473 (-46.196~27.250) | 0.605 | 1.114 |

Supplement 2-2. Coefficient of determination, R^2^=0.497 (*p*=0.002) (n=52). *CI* Confidence interval, *VIF* Variance inflation factor.

^a^ Co-ingestion: overdose with substances that delayed gastric emptying or induced hepatic enzymes.

Supplement 2-3. Multicollinearity between the variables on the first APAP concentration of the patients who did not receive AC treatment

|  | Unstandardized Coefficients (95% CI) | *p*-value | VIF |
| --- | --- | --- | --- |
| Intentionality | -13.570 (-69.879~42.740) | 0.629 | 1.244 |
| Weight (kg) | -0.402 (-1.914~1.110) | 0.591 | 1.169 |
| Ingested dose per kilogram of weight (mg/kg) | 0.392 (0.224~0.560) | <0.001 | 1.290 |
| Time from ingestion to the first concentration test (minute) | -0.062 (-0.142~0.019) | 0.130 | 1.158 |
| Acute starvation | -34.191 (-102.989~34.607) | 0.321 | 1.163 |
| Chronic liver disease | 44.288 (-68.271~156.848) | 0.431 | 1.081 |
| Chronic alcohol consumption | -14.768 (-73.200~43.664) | 0.613 | 1.095 |
| Co-ingestion^a^ | 22.207 (-27.166~71.580) | 0.369 | 1.123 |
| Albumin (g/dL) | -10.921 (-46.698~24.857) | 0.541 | 1.100 |

Supplement 2-3. Coefficient of determination, R^2^=0.490 (*p*<0.001) (n=52). *CI* Confidence interval, *VIF* Variance inflation factor.

^a^ Co-ingestion: overdose with substances that delayed gastric emptying or induced hepatic enzymes.
